# A risk signature of ubiquitin-specific protease family predict the prognosis and therapy of kidney cancer patients

**DOI:** 10.1186/s12882-023-03215-0

**Published:** 2023-05-31

**Authors:** Renjie Wang, Yang Liu, Jingxian Li, Yubao Zhao, Rui An, Zhifang Ma

**Affiliations:** 1 Department of Urology, Sixth Hospital of Shanxi Medical University, General Hospital of Tisco, Taiyuan, China; 2 The Second Hospital of Tianjin Medical University, Tianjin, China; 3 Department of Urology, First Hospital of Shanxi Medical University, Taiyuan, China

**Keywords:** USPs, Kidney cancer, Prognostic prediction, Targeted therapy

## Abstract

Ubiquitin-specific proteases (USPs) are closely related to protein fate and cellular processes through various molecular signalling pathways, including DNA damage repair, p53, and transforming growth factor-β (TGF-β) pathways. In recent years, increasing evidence has revealed the pivotal role of ubiquitination in tumorigenesis of KIRC. However, USPs' molecular mechanism and clinical relevance in kidney cancer still need further exploration. Our study first determined prognosis-related ubiquitin-specific proteases (PRUSPs) in KIRC. We found these genes co-expressed with each other and might regulate different substrates. Based on the USPs' expression, the PRUSPs risk signature was constructed to predict the survival probability of KIRC patients. The patients in high-PRUSPs-risk group showed a low survival rate. ROC and calibration curve indicated a discriminate capacity of the signature, and uni-/multi-variate Cox regression analysis revealed that the PRUSPs score is an independent prognostic factor. In different KIRC clinical subgroups and external validation cohorts (including E-MTAB-1980 and TCGA-KIRP cohorts), the PRUSPs risk signature showed strong robustness and practicability. Further analysis found that high-risk group showed activation of immune-related pathways and high PD-1/CTLA4 expression, revealing that high-risk patients might be sensitive to immunotherapy. In summary, we constructed the USPs risk signature to predict kidney cancer prognosis, which provided the theoretical foundation for further clinical or pre-clinical experiments.

**Supplementary Information:**

The online version contains supplementary material available at 10.1186/s12882-023-03215-0.

## Introduction

Renal cell carcinoma (RCC) is one of the most frequently diagnosed cancer in human beings [1], including several pathological subtypes. KIRC accounts for about 70% [2, 3]. Although KIRC could be successfully treated by surgery or ablative strategies, some patients will develop recurrence or metastases [4]. For recurrent or metastatic KIRC patients, medication (including chemotherapy, targeted therapy, and immunotherapy) is the primary approach. However, the clinical effect of these therapeutic methods was frustrating, as several targeted drugs (such as cytokines-high dose interleukin 2) showed a low response in clinical to treat KIRC patients [4]. Therefore, the clinical treatment of advanced KIRC still is one of the most challenging problem.

Under physiological circumstances, ubiquitylation and deubiquitylation are reversible processes of protein post-translation modification involving protein degradation regulation [5]. Balance dysfunction of the protein modification is closely related to tumorigenesis and other pathologies such as infection, inflammatory disorders, autoimmunity, etc. [6]. A series of proteases can remove ubiquitin modification, called deubiquitinases (DUBs). Ubiquitin-specific-processing proteases (USPs) are the largest subfamily of DUBs, which include 54 USPs so far [7]. Studies have shown that USPs are involved in multiple cancer-related pathways, such as protein kinase B (Akt), G protein-coupled receptor (GPCR), P53 pathways, DNA damage repair, etc. [8–14]. USPs are considered to play an important role in renal carcinoma. USP53 could regulate NF-kB pathway and inhibit KIRC proliferation and metastasis [15]. USP13 mediates the deubiquitination of ZHX2 and promotes tumorigenesis [16]. USP7 altered cell cycle G1/S phases and regulated renal cancer cell proliferation by targeting ARMC5 [17]. Several USPs have been used to develop inhibitors for cancer therapy [18]. However, whether these USPs could give rise to the potential therapeutic targets in KIRC or not still need further deeply exploration. Our research aimed to explore the concrete function and mechanism of USPs in KIRC and provide the theoretical foundation for further novel therapeutic targets exploration, pre-clinical trials, or clinical trials.

In this study, we first identified the differential expression genes and further determined the USPs correlated with the prognosis of KIRC patients, called PRUSPs. Then we constructed the PRUSPs risk signature, and the risk signature could independently predict the survival outcome of KIRC patients. The high PRUSPs risk group showed a worse outcome than the low PRUSPs risk group. We used the external validation cohort (E-MTAB-1980 and TCGA-KIRP) to perform the validation further. The results showed that the risk signature is practical in predicting RCC patients' prognosis. In addition, we found immune-related pathways and biological progress showed a higher enrichment in the high PRUSPs risk group. The mRNA expression of immune checkpoint (PD1 and CTLA4) was also up-regulated in the high PRUSPs risk group, indicating that the patients in high-risk group may be immune-activated, and the PRUSPs risk signature could identify a hot tumor. We also analyzed the potential targeting drugs between the high-/low-PRUSPs-risk group. The results showed that the patients in the high-PRUSPs-risk group might be sensitive to GDC-0449, SB590885, Embelin, AZD6244, Midostaurin, and sunitinib. Our study revealed the importance of PRUSPs in KIRC and identified the different clinical subgroups to identify hot/cold tumors and targeting drugs, which provided the theoretical basis for further targeting and immune therapy.

## Methods

### Public data acquisition

Based on the previous literature, 54 ubiquitin-specific proteases (USPs) were identified [7] (Supplementary Table 1). The mRNA expression profile (TCGA-KIRC, TCGA-KIRP, and E-MTAB-1980) datasets were downloaded from the public database (https://xena.ucsc.edu/; https://www.ebi.ac.uk/arrayexpress). The normalized mRNA data of GSE11151 was downloaded from the GEO database (https://www.ncbi.nlm.nih.gov/gds). The mRNA expression levels of the TCGA-KIRC and TCGA-KIRP datasets were normalized and transformed into LCPM units using the edgeR package. The human protein atlas (HPA) website (http://www.proteinatlas.org/), including the immunohistochemistry data, was used for validation.

### Differential expression analysis

The limma R package downloaded from the Bioconductor (https://www.bioconductor.org/) was applied to screen DEGs. The *p*-value was adjusted by the method—Benjamini Hochberg. A filtering threshold of the DEGs was absoluted Log2(Fold change) > 0.5 and adjusted *p*-value < 0.05.

### Correlation between PRUSPs

The *corAndPvalue* function from the WGCNA R-package was used to calculate the Pearson correlation coefficient (PCC) between PRUSPs in mRNA levels. The *P*-value was adjusted by the FDR method. Then we applied the string website (https://string-db.org/) to analyze the protein–protein interaction (PPI) of these PRUSPs.

### Construction of PRUSPs risk signature

Through the “*createDataPartition*” R-function, the KIRC patients were evenly separated into two cohorts (including training and testing cohorts) at a ratio of 1:1. Basing on the *glmnet* R-package, the training cohort was used to construct a prognostic risk signature utilizing the Lasso-penalized Cox regression analysis. Then we constructed a PRUSPs risk score, and the risk score was applied to validate using testing and external validation cohort.

### Nomogram construction

The variables (including gender, PRUSPs risk score, age, grade, and stage) were matched with clinical outcomes to conduct univariate Cox regression. Then the prognosis-related variables were subjected to the multivariate Cox regression analysis to explore the dependent prognostic variables. Then the stepwise regression method was applied to screen variables further based on the minimum of the Akaike information criterion (AIC). A nomogram was constructed to estimate the survival probability of 3, 5, and 7 years. ROC curve, calibration curve, and decision curve analysis (DCA) were used to estimate the clinical benefits and discriminative accuracy of the nomogram model.

### Mechanisms, drug predictions, and immune infiltration analysis

Based on the median value of the PRUSPs risk score, the patients were divided into high-/low-risk groups. Then the DEGs (|LogFC|> 0.5 and adjusted. *P*.value < 0.05) between high/low-risk groups were subjected to the clusterProfiler R package. The top 5 terms of KEGG pathways [19–21], cell components (CC), molecular functions (MF), and biological progressions (BP) are shown. Then the DEGs were subjected to the public drug prediction website—designN (https://design-v2.cancerresearch.my/query). Potential prediction inhibitors are generated. The connectivity score was calculated to demonstrate drug sensitivity. A score closer to 1 suggests that the drug is more effective for the patients in high-risk groups. Furthermore, the 24 immune cell gene sets were acquired from the published research [22] to calculate the immune infiltration levels by utilizing the single-sample gene set enrichment (*ssGSEA*) method of the *GSVA* R-package. Appling *CIBERSORT* methods calculated the 22 immune cells’ proportion.

### Statistical analysis

We used the unpaired t-test to analyze the median value difference between the two groups. The univariate and multivariate Cox regression analyses were used to screen the independent prognostic factors. The log-rank test was used to examine the significance of the uni-/multivariate Cox regression analysis. The Pearson method was used to assess the correlation between the expression value of two genes.

## Results

### Expression alteration of USPs in KIRC

**Fig. 1:**
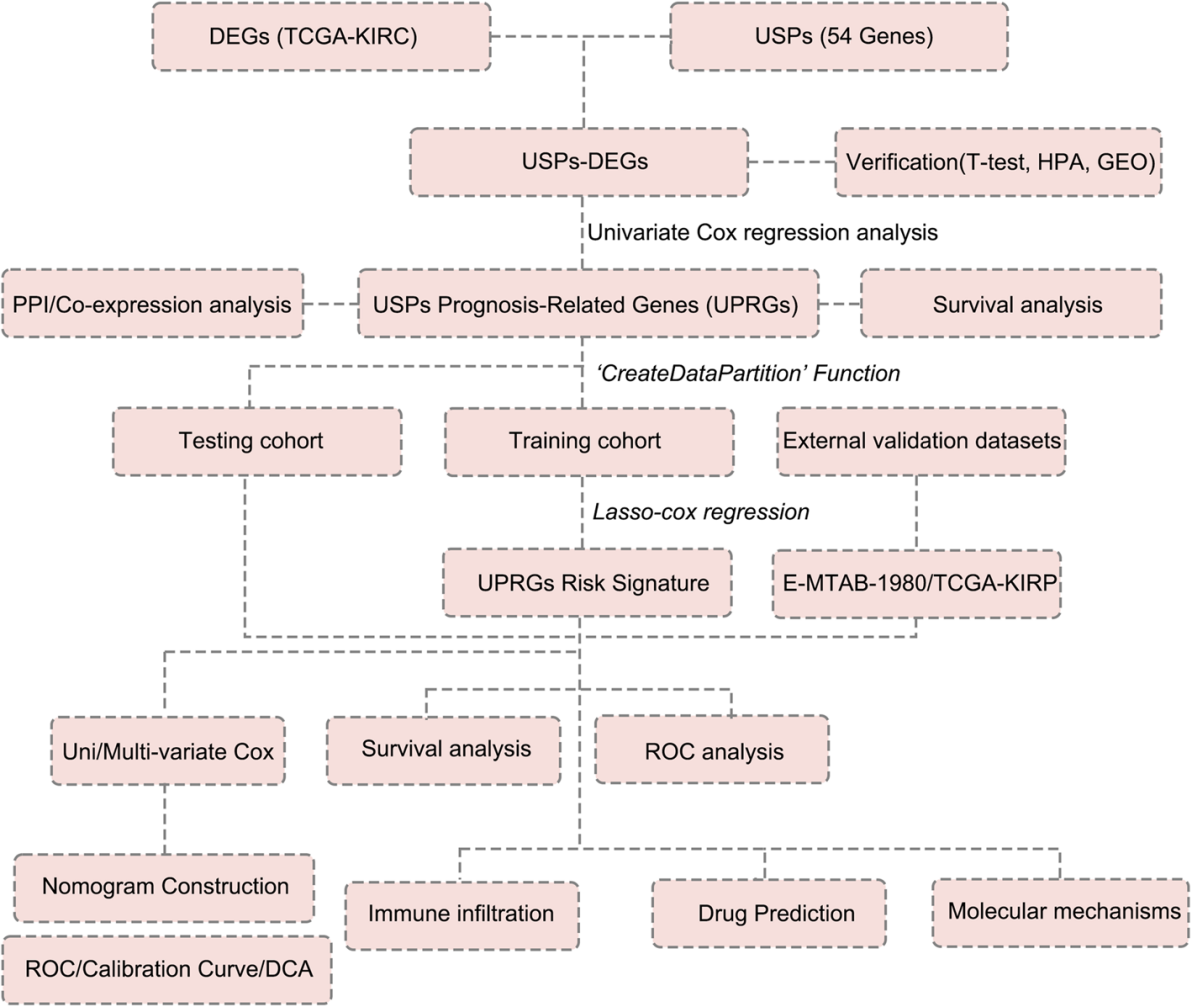
Flow chart of the study

**Fig. 2:**
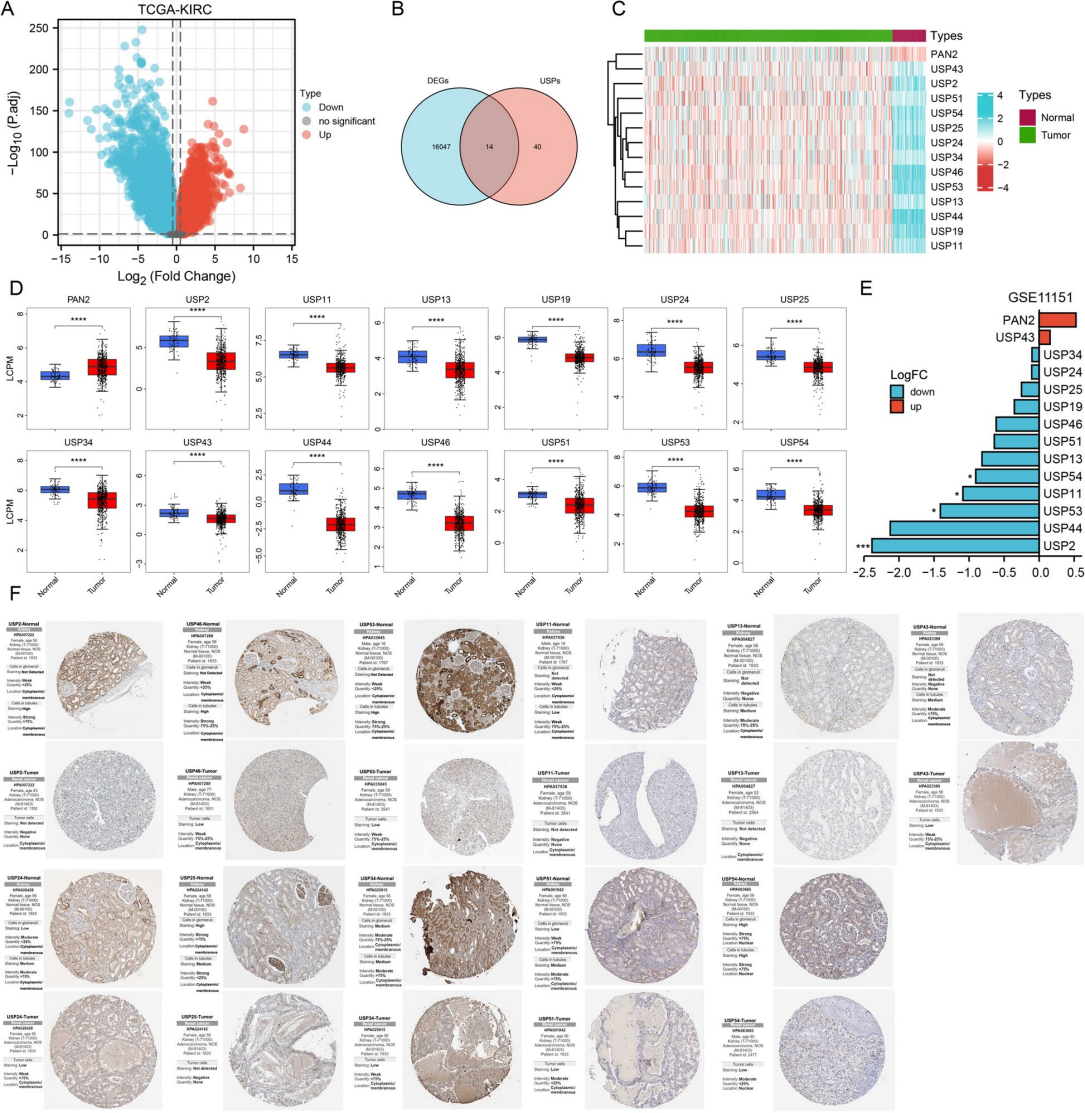
Expression alteration of USPs family. **A** The volcano plot showed the DEGs of KIRC. **B** The Venn diagram showed the overlapping genes between USPs and DEGs. **C** The heatmap showed differential expression USPs in KIRC-tumor and normal tissues. **D** The boxplots show the mRNA expression of 14 USPs in the TCGA-KIRC tumor and normal tissues. The significance level between the tumor and normal tissues was analyzed by t-test. **E** The bar plot showed the expression alteration of 14 USPs in GSE11151-KIRC. **F** Immunohistochemistry of the USPs in KIRC and normal tissues. *P-value < 0.05, **P-value < 0.01, ***P-value < 0.001, ****P-value < 0.0001

The workflow of our study is shown in Fig. 1. To determine the differential expression USPs, we first calculated the differential expression genes (DEGs) between TCGA-KIRC and adjacent normal tissues (Fig. 2A). 16,061 DEGs were determined. Among these, 14 USPs showed differential expression in KIRC and could distinguish tumor and normal tissues well (Fig. 2B-C). PAN2 showed up-regulation, USP2, USP11, USP13, USP19, USP24, USP15, USP34, USP43, USP44, USP46, USP51, USP53, and USP54 represented down-regulation in KIRC (Fig. 2D). We used the external dataset (GSE11151) to conduct differential expression verification; the expression alteration of USPs was consistent with TCGA-KIRC except for USP43 (Fig. 2E). In the HPA database, we found that the protein levels of USP2, USP46, USP53, USP11, USP24, USP25, USP34, and USP51 were higher in normal tissues compared to tumor tissues. The protein levels of USP13 and USP43 showed a medium staining intensity in tubules but were not detected or low staining intensity in tumor tissues. However, other genes were not found in the HPA database (Fig. 2F).

### Clinical relevance and cross-talk of USPs

**Fig. 3:**
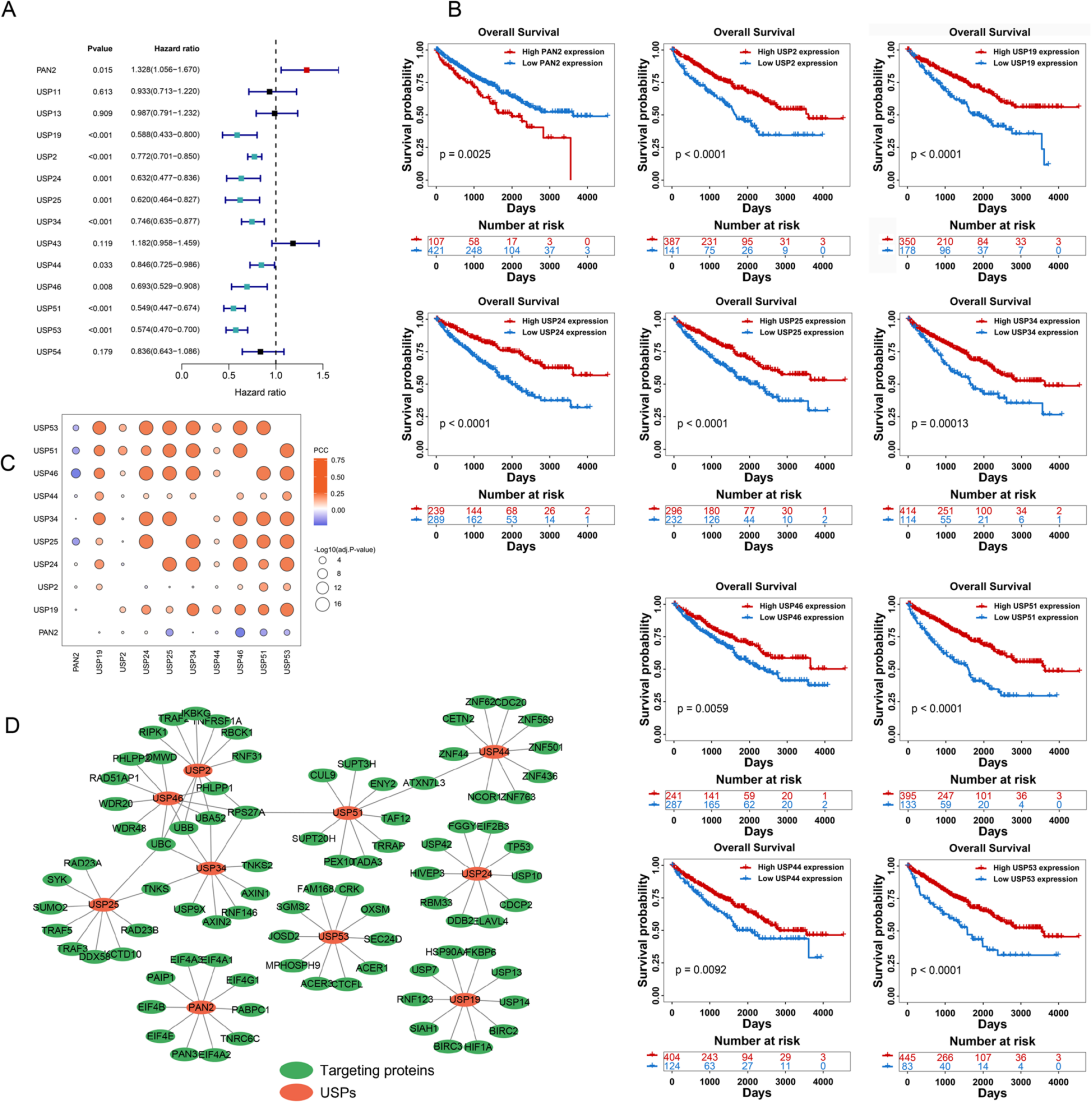
Clinical relevance and co-expression analysis of USPs. **A** The prognosis-related USPs were determined by univariate Cox regression analysis and demonstrated using the forest plots. **B** The Kaplan–Meier survival curves showed prognosis of high-/low-USPs expression groups of TCGA-KIRC. The log-rank test was used to estimate the significance. **C** Co-expression analysis of prognosis-related USPs. **D**, Protein–protein interaction network of prognosis-related USPs

We performed the univariate Cox regression analysis to determine the prognosis-related USPs (PRUSPs). 10 USPs were correlated with the survival of the KIRC patients, including PAN2 (HR = 1.328, *P*-value = 0.015), USP19 (HR = 0.588, *P*-value < 0.001), USP2 (HR = 0.772, *P*-value < 0.001), USP24 (HR = 0.632, *P*-value = 0.001), USP25 (HR = 0.620, *P*-value = 0.001), USP34 (HR = 0.746, *P*-value < 0.001), USP44 (HR = 0.846, *P*-value = 0.033), USP46 (HR = 0.693, *P*-value = 0.008), USP51 (HR = 0.549, *P*-value < 0.001), and USP53 (HR = 0.574, *P*-value < 0.001) (Fig. 3A). High PAN2 expression showed a worse outcome. Other USPs (including USP2, USP19, USP24, USP25, USP34, USP44, USP46, USP51, and USP53) represent the protective factors for KIRC patients’ outcomes. In the low expression group of these USPs, the KIRC patients showed poor outcomes (Fig. 3B). In addition, we found that PAN2 showed a negative correlation or no correlation with other PRUSPs in mRNA expression level, and other PRUSPs showed a positive correlation with each other, indicating that PAN2 might play a different biological function in KIRC (Fig. 3C). We further analyzed the protein–protein interaction (PPI) for PRUSPs. We observed that some PRUSPs directly interacted with the same proteins, such as USP46 and USP2, interacted with PHLPP1, UBB, and UBC. USP44 and USP53 interacted with ATCN7L3. PAN2, USP53, USP24, and USP19, did not show the same protein interaction with other PRUSPs (Fig. 3D). These results reveal that they might play different roles by regulating different substrates in KIRC.

### Construction of PRUSPs risk signature

**Fig. 4:**
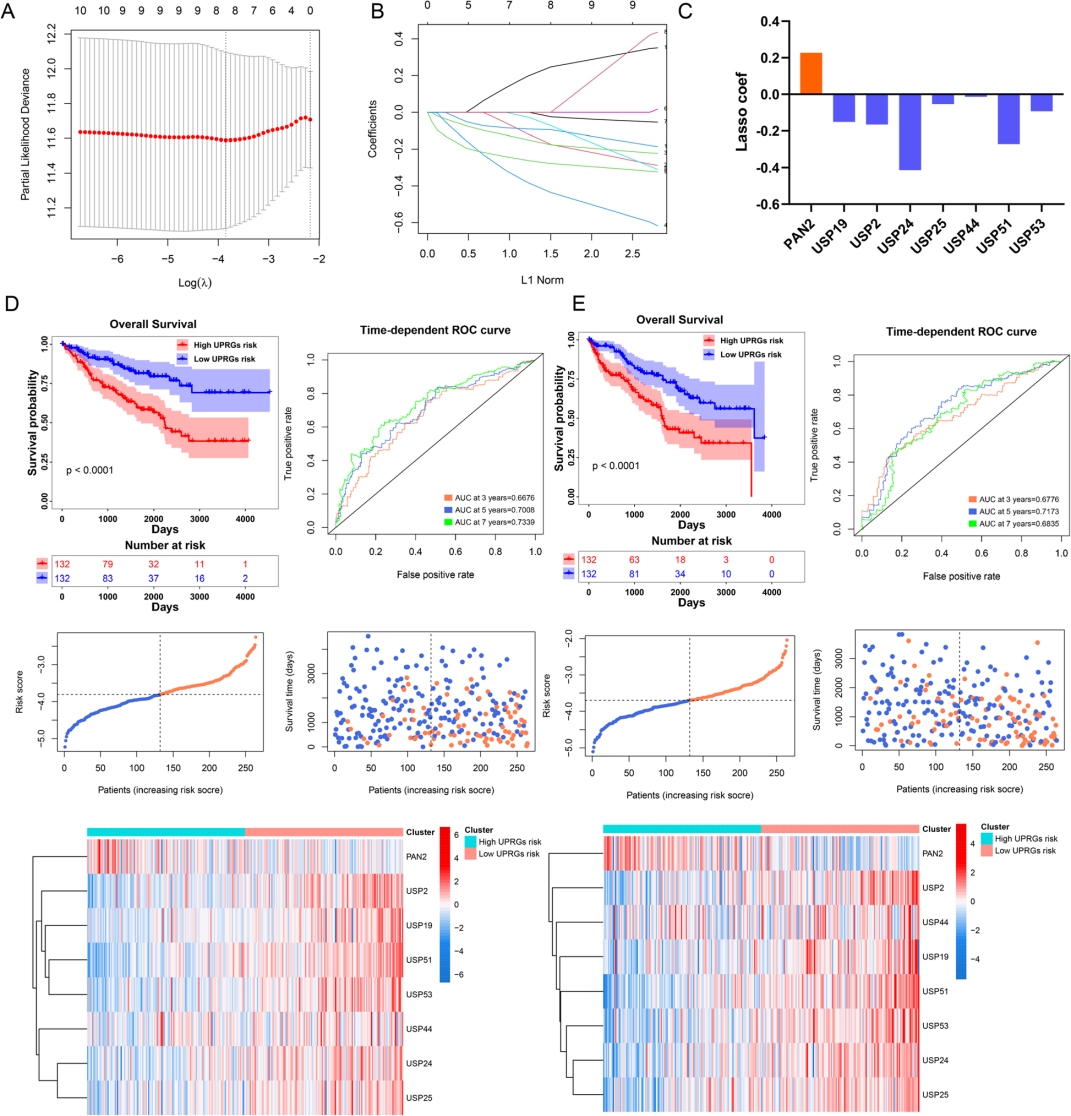
Construction of USPs risk signature. **A** and **B** The progressions of lasso-cox regression analyses progression. **C** The lasso-cox regression coefficients of USPs are shown by bar plot. **D** and **E** The Kaplan–Meier curves of high-/low-PRUSPs-risk groups in the TCGA-KIRC training/testing cohort; AUC value of time-dependent ROC curves demonstrate the prognostic predicted capacity of PRUSPs risk signature. The dot-plot was depicted to represent the OS, OS status, and risk score in the TCGA-KIRC training/testing cohort; the heatmap showed the expression of PRUSPs in high and low-risk groups of the training/testing cohort

To further determine the contribution of these PRUSPs to KIRC patients, we constructed a PRUSPs signature utilizing LASSO Cox regression analysis. The training cohort was used to construct the risk signature for OS of KIRC patients based on the mRNA expression of 10 PRUSPs (Fig. 4A-B). 8 PRUSPs signature were finally determined (Fig. 4C). The equation of the risk score was determined as follows:

$$Risk score = 0.22739298*PAN2-0.15106386*USP19-0.16540585*USP2-0.41409702*USP24-0.05350950*USP25-0.01392374*USP44-0.27152405*USP51-0.09273768*USP53$$

Then the training cohort was divided into two groups (high PRUSPs-risk group and low PRUSPs-risk group) according to the median value. We found that the patients in the high PRUSPs-risk group showed a worse prognosis than those in the low PRUSPs-risk group (Fig. 4D). The time-dependent AUC showed a discriminative accuracy in 3-/5-/7-years survival (3 years = 0.6676, 5 years = 0.7008, and 7 years = 0.7339). In addition, the number of dead patients increased accompanied by the increasing PRUSPs risk score. Interestingly, we found PAN2 showed a higher expression in the high PRUSPs risk group. In contrast, other PRUSPs, showed a higher expression in low PRUSPs risk groups. We conducted the same analysis in the KIRC testing and external validation cohorts (E-MTAB-1980 and TCGA-KIRP). The results represent a feasible prediction capacity of the PRUSPs risk signature (Fig. 4E, Fig. S1A-B).

### Robustness analysis and nomogram construction

**Fig. 5:**
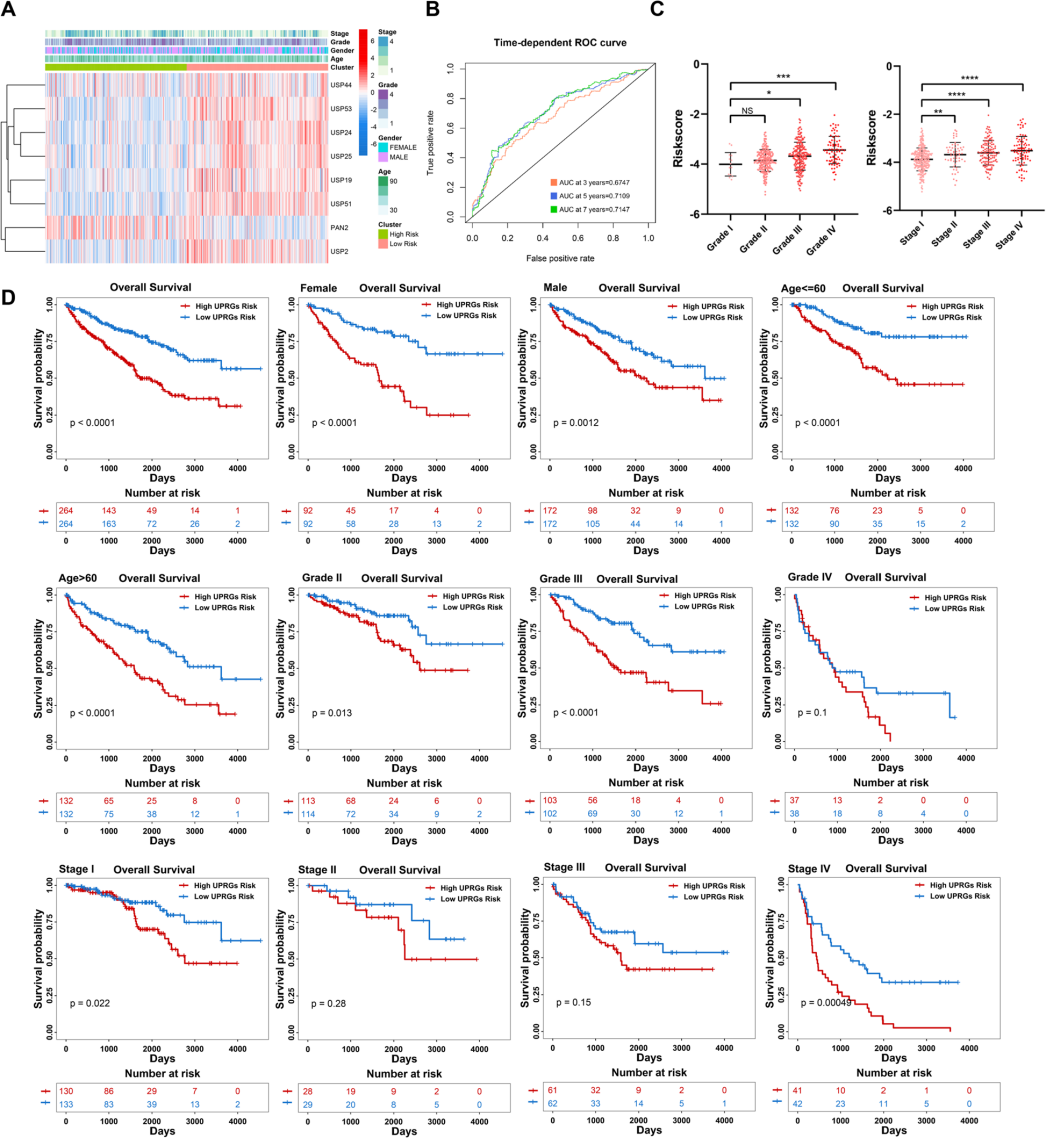
Prediction capacity analysis. **A** and **B** The expression heatmap of PRUSPs and ROC curve based on the PRUSPs risk signature. **C** Correlation of PRUSPs risk score, pathological stage and histological grades. **D** The Kaplan–Meier survival analysis validates the PRUSPs-risk signature in different clinical subgroups of TCGA-KIRC. *P-value < 0.05, **P-value < 0.01, ***P-value < 0.001, ****P-value < 0.0001

**Fig. 6:**
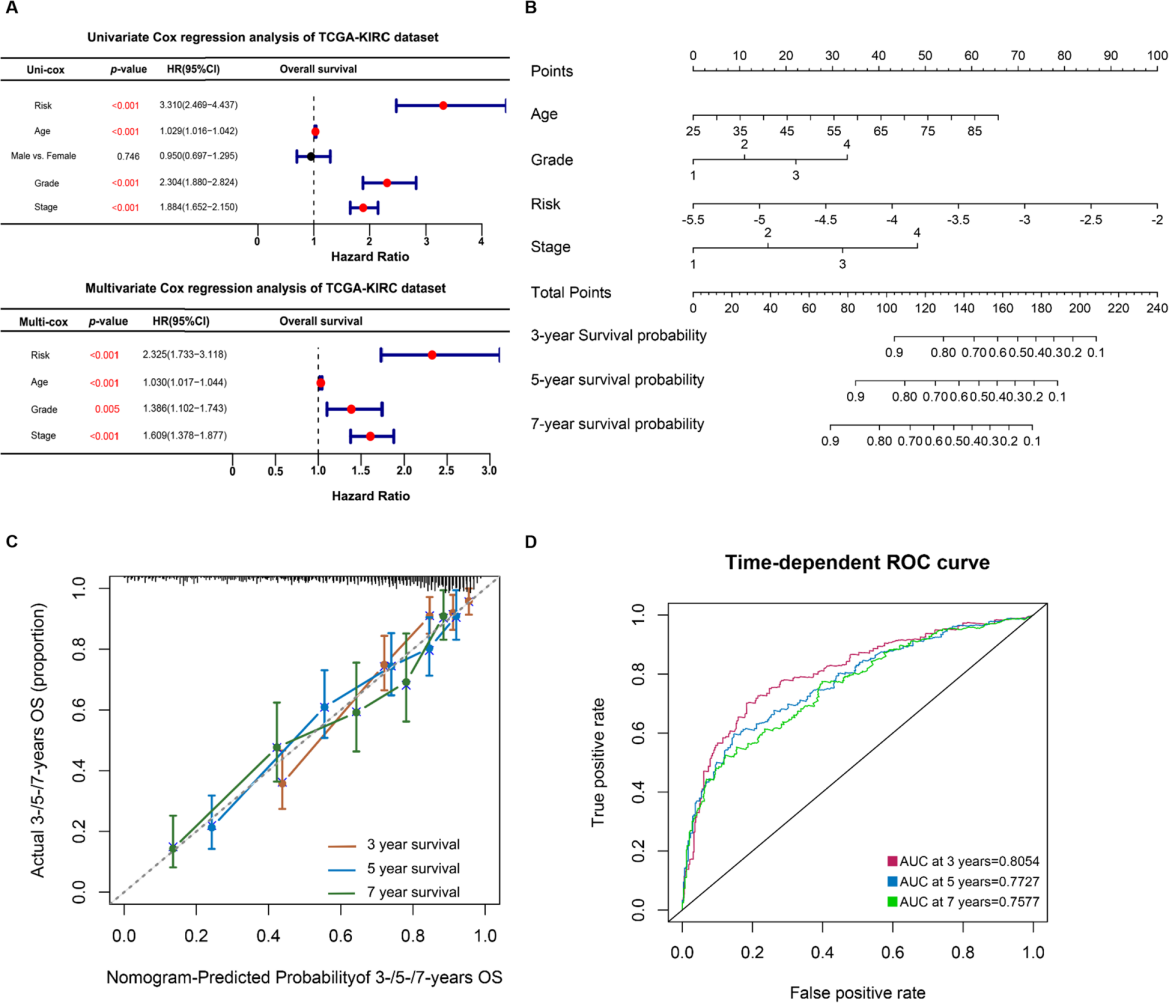
Nomogram model construction. **A** Univariate/multivariate cox regression analysis showed the prognosis-related variables, including risk score, age, gender, grade, and stage. **B** Nomogram construction is based on clinical variables, including age, grade, stage, and PRUSPs-risk score. **C** and **D** Calibration and ROC curve assess the prediction capacity of PRUSPs risk signature. *P-value < 0.05, **P-value < 0.01, ***P-value < 0.001, ****P-value < 0.0001

In the whole KIRC cohort, PAN2 expression was higher, and other PRUSPs' expressions were lower in the high PRUSPs-risk cohort (Fig. 5A). The time-dependent AUC showed a discriminative accuracy based on the ROC analysis (3 years = 0.6747, 5 years = 0.7109, and 7 years = 0.7147) (Fig. 5B). The tumor grade and stage gradually increased with increasing risk, indicating that the risk score could predict tumor progression (Fig. 5C). Furthermore, the patients with higher PRUSPs risk showed a worse outcome in different clinical subgroups. These results showed that the PRUSPs risk signature possesses stronger robustness for predicting KIRC patients (Fig. 5D). We further explore whether the PRUSPs risk signature was affected by other clinical signatures, such as age, gender, grade, and stage. In the univariate Cox regression analysis, the PRUSPs risk signature (HR = 3.310, *P*-value < 0.001), age (HR = 1.029, *P*-value < 0.001), histological grade (HR = 2.304, *P*-value < 0.001), and pathological stage (HR = 1.884, *P*-value < 0.001) significantly affect the prognosis of the KIRC patients (Fig. 6A). We further utilized these four variables to perform the multivariate Cox regression analysis. These results showed that these four variables were also correlated with the patient's prognosis (For riskscore: HR = 2.325, *P*-value < 0.001; age: HR = 1.030, *P*-value < 0.001; Grade: HR = 1.386, *P*-value = 0.005; Stage: HR = 1.609, *P*-value < 0.001), indicating that these four variables were the independent prognostic factors for KIRC patients. Subsequently, a nomogram model based on these four variables was constructed to predict the survival probability of 3-/5-/7-years of KIRC patients (Fig. 6B). The calibration curve and time-dependent AUC indicated that the model possesses a discriminative accuracy for predicting 3-/5-/7-years overall survival (Fig. 6C and D). Moreover, The model showed the best clinical application value compared with other variables based on the DCA plots (Fig. S2). Overall, our PRUSPs risk signature possesses stronger robustness and independently predicts the prognosis of KIRC patients.

### Mechanisms, drug prediction, and immune infiltration in high-/low risk groups

**Fig. 7:**
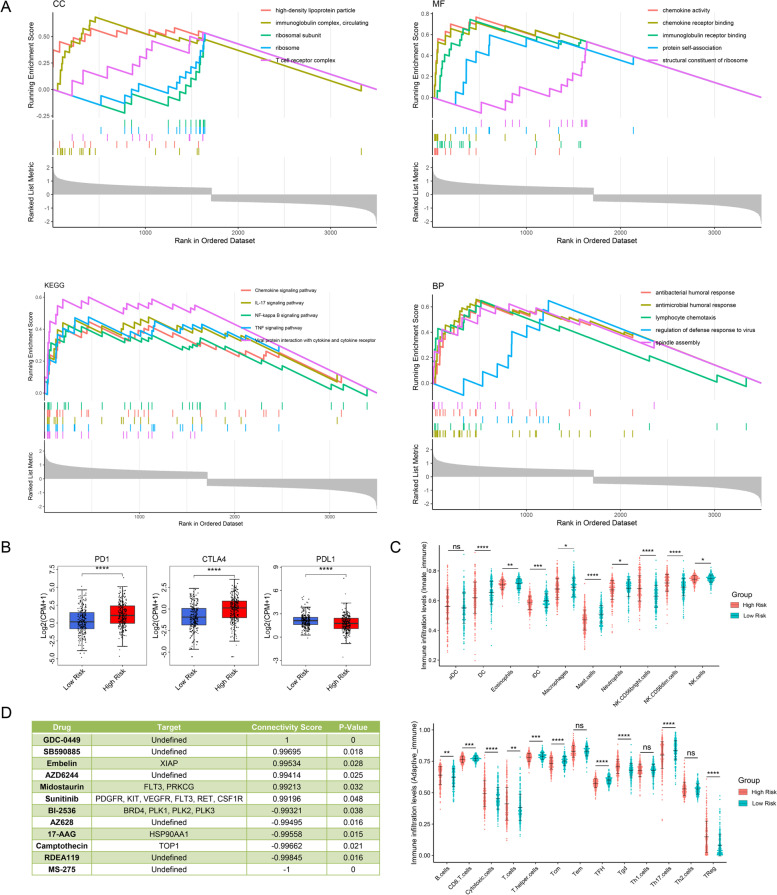
Functional enrichment analysis and drug prediction. **A** GSEA enrichment analysis between high-/low-PRUSPs-risk groups. **B** The mRNA expression of immune checkpoint in high-/low-risk groups. **C** The immune infiltration levels between the high-/low-risk group. **D** Targeted drug prediction between high-/low-PRUSPs-risk groups. *P-value < 0.05, **P-value < 0.01, ***P-value < 0.001, ****P-value < 0.0001

The differential clinical outcomes in high-/low-PRUSPs risk groups drive us to explore the potential mechanisms. We subjected the DEGs between high-/low-PRUSPs risk groups to *clusterProfiler* R-package and analyzed the enrichment situation of the KEGG pathways and gene ontology, including cell components (CC), biological progressions (BP), and molecular functions (MF). We found several terms of CCs (including high-density lipoprotein particle, immunoglobulin complex, circulating, ribosomal subunit, ribosome, and T cell receptor complex), MFs(including chemokine activity, chemokine receptor binding, immunoglobulin receptor binding, protein self-association, and structural constituent of the ribosome), BPs (antibacterial humoral response, antimicrobial humoral response, lymphocyte chemotaxis, regulation of defense response to virus, and spindle assembly), and KEGG (chemokine signaling pathway, IL-17 signaling pathway, NF-kappa-B signaling pathway, TNF signaling pathway, and viral protein interaction with cytokine and cytokine receptor) differentially enriched in high-/low-PRUSPs risk groups, indicating that immune system dysfunction influences the prognosis of KIRC patients (Fig. 7A). We further investigated the expression of three immune checkpoints in high-/low-PRUSPs risk groups. CTLA4 and PD1 showed a higher expression in high-risk groups than in low-risk groups (Fig. 7B). In addition, multiple immune cell activity was higher in high PRUSPs risk groups, such as NK cells, B cells, cytotoxic cells, T cells, Treg cells, etc. (Fig. 7C). These results suggest that the patients in high PRUSPs risk groups might be sensitive to immune therapy based on anti-PD1. Moreover, targeted therapy is also one of the treatment methods for metastatic renal cell carcinoma. We further subjected the DEGs between high and low PRUSPs risk groups to the public website—designN to predict the potential targeting drugs. The results showed that the patients in the high-PRUSPs-risk group might be sensitive to the GDC-0449, SB590885, Embelin, AZD6244, midostaurin, and sunitinib (Fig. 7D). All in all, our research feasibly stratified the KIRC patients into different subgroups based on the expression of USPs and predicted patient outcomes and potential treatment modalities.

## Discussion

For recurrent or advanced KIRC, medicine is the primary approach in clinical treatment, such as immunotherapy, targeted therapy, and chemotherapy [4]. The surgical approaches are generally curative when the tumor is confined to the kidney. However, one-third of KIRC might present in metastases or recurrence, and patients treated with traditional medicine showed a low survival probability [23]. Therefore, novel treatments for metastatic KIRC still need to develop and explore. Studies have indicated that USPs are pivotal for cancer progression, and several USPs have been utilized as targets to develop inhibitors for cancer prevention [18, 24, 25]. ML364 is a small molecule inhibitor of the deubiquitinase USP2, which could degrade CCND1 and prevent cancer cell cycle progression in colorectal cancer [26]; USP7 could induce apoptosis in multiple myeloma cells resistant to conventional and bortezomib therapies [27]; Developing the medicines based on the USPs are considered as a potential new treatment for cancer. Research have been reported on the role of USPs in KIRC progression [15–17, 28]. USP13 mediates the deubiquitination of ZHX2 and promotes tumorigenesis in kidney cancer [29]. The silencing of USP39 could block the activation of Akt signaling pathways and markedly suppress RCC cell proliferation and invasion [30]. USP7 altered cell cycle G1/S phases and regulated renal cancer cell proliferation by targeting ARMC5 [17]. However, as a large subfamily of DUBs, many mechanisms or unknown functions of USPs in KIRC need to be explored. Of note, due to different responses to current medicine, it is necessary to construct a feasible stratification of KIRC patients to determine the subgroups which might benefit from one or some drugs.

Our study retrospectively analyzed the mRNA expression profile and clinical data from the TCGA, GEO, and Arrayexpress databases. We first screened the USPs which might play an essential role in KIRC. Among these, PAN2 is up-regulated and acts as a risk factor in KIRC overall survival (OS). Other USPs, including USP19, USP2, USP24, USP25, USP34, USP44, USP46, USP51, and USP53, are down-regulated and act as protective factors for KIRC OS. Of note, these analyses only showed the association between genes and prognosis, but not a causal relationship. USP19, USP44, USP46, and USP53 has been revealed as potential tumor suppressor in KIRC in the previous study [15, 31–33]. Interestingly, these USPs were down-regulated in KIRC and prevented patients from dying. Recovery from the expression of these USPs might play a role in tumor suppression. However, few reports about the function of other USPs, exploring the potential function of these USPs in KIRC is also necessary.

An eight-USPs risk signature was constructed by the LASSO-Cox regression analysis. In the TCGA-KIRC dataset, the patients with high-PRUSPs-risk signatures showed a worse prognosis than that with low-PRUSPs-risk signatures. We used two external validation (E-MTAB-1980 and TCGA-KIRP) datasets to verify the risk signature's practicability further, and the results showed that the risk signature possesses a discriminate capacity. For kidney cancer, the most common type is KIRC, which represents up to 70%, and KIRP, which represents up to 15% [34, 35]. Therefore, our risk signature might be suitable for KIRC and could be extended to predict the prognosis of KIRP simultaneously. In addition, we found that the PRUSPs-risk signature is an independent prognostic factor, indicating that the risk signature is not affected by other clinical variables.

A previous study indicated that the patients' different responses to medicine were due to different subtypes [36]. Therefore, we are trying to explore potential treatments by mining molecular mechanisms. Through the functional enrichment analysis, we observed that multiple immune-related pathways showed high enrichment in high-risk group, such as the T cell receptor complex, TNF signaling pathway, and chemokine receptor. T cell receptor-based cancer immunotherapy is an emerging efficacy [37], which provide many advantages to other immune therapy because it can target the vast repertoire of mutated and cancer-associated protein found in all location of subcellular. TNF also is an important immunotherapeutic target [38]. Researchers indicated that removing soluble TNF-alpha receptors local enhancement of endogenous TNF-alpha activity may provide for enhanced tumor cell death. Chemokines and chemokine receptors are expressed by both tumor cells and leukocyte infiltrate. It is reported that targeting the overexpressed chemokine receptors could kill tumor cells [39]. These findings suggested that the patients in high-risk group might benefit from the different immunotherapy-based therapeutic targets. Further analysis revealed that the patients in high PRUSPs risk group showed a higher expression of PD-1 and CTLA-4. For some kidney cancer patients, anti-PD1 therapy demonstrated durable tumor control [40]. Recent research even indicated that the combination of PD-1 and CTLA-4 blockade was more effective than either agent alone in melanoma, highlighting the need for biomarkers for combination regimens [41]. On the basis of the successes of antibodies targeting CTLA4 and PD1, multiple novel immunotherapies are now in clinical development for RCC patients [42]. Therefore, we speculated that the high-PRUSP risk group's patients could benefit from the combination of PD-1 and CTLA-4 blockade, which needs further clinical experiment validation. Currently, sunitinib is a commonly used first-line target drug for mRCC [43–45], which could prolong the progression-free survival (PFS) of mRCC patients. However, many patients develop progression after sunitinib treatment and cannot help changing other medicines [46]. Therefore, not all patients respond to target drugs; specific patients need to treat for specific drugs, which is relied on the specific subtypes based on the biomarker. In our research, we predicted that the patients in the high-PRUSPs-risk group might be sensitive to sunitinib. Therefore, this type of patient might benefit from sunitinib treatment, whereas for the patients in the low-PRUSPs-risk group, the treatment strategy should be transformed based on the treatment effect. In addition, we found that other drugs might be used to treat the patients in high-PRUSPs-risk groups, such as GDC-0449, SB590885, Embelin, AZD6244, and Midostaurin. This result provides the treatment chosen for the condition of failure of the treatment of the first line. What’s more, we noticed that only PAN2 was highly expressed in high-PRUSPs-risk group. A previous study reported that the deletion of PAN2 could sensitize cells to PARP inhibition in vitro and in vivo [47]. PAN2 silencing or developing correspondent inhibitors combined with sunitinib may have more significant clinical benefits for the patients in high-PRUSPs-risk group. Recovering the expression of the tumor suppressor, such as USP19, USP44, and USP53, also might reach the tumor-inhibited effect for this type of patient.

In conclusion, our study revealed several USPs that might play important roles in KIRC and established the novel USPs risk signature to predict the prognosis of KIRC patients. Of note, there are still some limitations that need to the discussion. The function of several USPs that have not been reported still needs verification by subsequent experiments. Moreover, whether the USPs risk signature is applicable for predicting clinical outcomes requires prospective studies for validation.

## Supplementary Information

**Additional file 1:** **Table S1.** Information of 54 ubiquitin-specific proteases.

**Additional file 2:** **Figure S1.** Validation of PRUSPs risk signature in external datasets. (A) The Kaplan-Meier survival curves of high-/low-PRUSPs-risk groups in the E-MTAB-1980 dataset. AUC value of time-dependent ROC curves demonstrates the prognostic capacity of PRUSPs risk signature. The dot-plot was depicted to represent the OS, OS status, and risk score in the E-MTAB-1980 dataset. The heatmap showed the expression of PRUSPs in high and low-risk groups of the E-MTAB-1980 dataset. (B) The Kaplan-Meier survival curves of high-/low-PRUSPs-risk groups in the TCGA-KIRP dataset. AUC value of time-dependent ROC curves demonstrates the prognostic capacity of PRUSPs risk signature. The dot-plot was depicted to represent the OS, OS status, and risk score in TCGA-KIRP dataset. The heatmap showed the expression of PRUSPs in high and low-risk groups of the TCGA-KIRP dataset. **Figure S2.** DCA analysis of different combination based clinical variables.

## Data Availability

The datasets presented in this study can be found in online repositories, including UCSC xena (https://xena.ucsc.edu/); Arraryexpress (https://www.ebi.ac.uk/arrayexpress); GEO database (https://www.ncbi.nlm.nih.gov/gds); HPA website (http://www.proteinatlas.org/); Bioconductor (https://www.bioconductor.org/); string website (https://string-db.org/); designN (https://design-v2.cancerresearch.my/query).

## References

[1] Capitanio U, Bensalah K, Bex A, Boorjian SA, Bray F, Coleman J, Gore JL, Sun M, Wood C, Russo P. Epidemiology of Renal Cell Carcinoma. Eur Urol. 2019;75(1):74–84. 10.1016/j.eururo.2018.08.036 PMC8397918 30243799

[2] Kajdasz A, Majer W, Kluzek K, Sobkowiak J, Milecki T, Derebecka N, Kwias Z, Bluyssen HAR, Wesoly J: Identification of RCC Subtype-Specific microRNAs-Meta-Analysis of High-Throughput RCC Tumor microRNA Expression Data. Cancers (Basel). 2021;13(3):548. 10.3390/cancers13030548 PMC7867039 33535553

[3] Jonasch E, Walker CL, Rathmell WK (2021). Clear cell renal cell carcinoma ontogeny and mechanisms of lethality. Nat Rev Nephrol.

[4] Jonasch E, Gao J, Rathmell WK (2014). Renal cell carcinoma. BMJ.

[5] Chen S, Liu Y, Zhou H: Advances in the Development Ubiquitin-Specific Peptidase (USP) Inhibitors. Int J Mol Sci. 2021;22(9):4546. 10.3390/ijms22094546 PMC8123678 33925279

[6] Popovic D, Vucic D, Dikic I (2014). Ubiquitination in disease pathogenesis and treatment. Nat Med.

[7] Chen D, Ning Z, Chen H, Lu C, Liu X, Xia T, Qi H, Wang W, Ling T, Guo X (2020). An integrative pan-cancer analysis of biological and clinical impacts underlying ubiquitin-specific-processing proteases. Oncogene.

[8] Huang TT, D'Andrea AD (2006). Regulation of DNA repair by ubiquitylation. Nat Rev Mol Cell Biol.

[9] Meulmeester E, Pereg Y, Shiloh Y, Jochemsen AG (2005). ATM-mediated phosphorylations inhibit Mdmx/Mdm2 stabilization by HAUSP in favor of p53 activation. Cell Cycle.

[10] Milojevic T, Reiterer V, Stefan E, Korkhov VM, Dorostkar MM, Ducza E, Ogris E, Boehm S, Freissmuth M, Nanoff C (2006). The ubiquitin-specific protease Usp4 regulates the cell surface level of the A2A receptor. Mol Pharmacol.

[11] Stevenson LF, Sparks A, Allende-Vega N, Xirodimas DP, Lane DP, Saville MK (2007). The deubiquitinating enzyme USP2a regulates the p53 pathway by targeting Mdm2. EMBO J.

[12] Xing C, Lu XX, Guo PD, Shen T, Zhang S, He XS, Gan WJ, Li XM, Wang JR, Zhao YY (2016). Ubiquitin-Specific Protease 4-Mediated Deubiquitination and Stabilization of PRL-3 Is Required for Potentiating Colorectal Oncogenesis. Cancer Res.

[13] Xu D, Shan B, Lee BH, Zhu K, Zhang T, Sun H, Liu M, Shi L, Liang W, Qian L (2015). Phosphorylation and activation of ubiquitin-specific protease-14 by Akt regulates the ubiquitin-proteasome system. Elife.

[14] Zhang D, Zaugg K, Mak TW, Elledge SJ (2006). A role for the deubiquitinating enzyme USP28 in control of the DNA-damage response. Cell.

[15] Gui D, Dong Z, Peng W, Jiang W, Huang G, Liu G, Ye Z, Wang Y, Xu Z, Fu J (2021). Ubiquitin-specific peptidase 53 inhibits the occurrence and development of clear cell renal cell carcinoma through NF-kappaB pathway inactivation. Cancer Med.

[16] Xie H, Zhou J, Liu X, Xu Y, Hepperla AJ, Simon JM, Wang T, Yao H, Liao C, Baldwin AS (2022). USP13 promotes deubiquitination of ZHX2 and tumorigenesis in kidney cancer. Proc Natl Acad Sci U S A.

[17] Yan G, Liu N, Tian J, Fu Y, Wei W, Zou J, Li S, Wang Q, Li K, Wang J (2021). Deubiquitylation and stabilization of ARMC5 by ubiquitin-specific processing protease 7 (USP7) are critical for RCC proliferation. J Cell Mol Med.

[18] Young MJ, Hsu KC, Lin TE, Chang WC, Hung JJ (2019). The role of ubiquitin-specific peptidases in cancer progression. J Biomed Sci.

[19] Kanehisa M, Goto S (2000). KEGG: kyoto encyclopedia of genes and genomes. Nucleic Acids Res.

[20] Kanehisa M (2019). Toward understanding the origin and evolution of cellular organisms. Protein Sci.

[21] Kanehisa M, Furumichi M, Sato Y, Kawashima M, Ishiguro-Watanabe M (2023). KEGG for taxonomy-based analysis of pathways and genomes. Nucleic Acids Res.

[22] Bindea G, Mlecnik B, Tosolini M, Kirilovsky A, Waldner M, Obenauf AC, Angell H, Fredriksen T, Lafontaine L, Berger A (2013). Spatiotemporal dynamics of intratumoral immune cells reveal the immune landscape in human cancer. Immunity.

[23] Weiss RH, Lin PY (2006). Kidney cancer: identification of novel targets for therapy. Kidney Int.

[24] Guo J, Zhao J, Fu W, Xu Q, Huang D (2022). Immune Evasion and Drug Resistance Mediated by USP22 in Cancer: Novel Targets and Mechanisms. Front Immunol.

[25] Turnbull A, Ioannidis S, Krajewski W, Pinto-Fernandez A, Heride C, Martin A, Tonkin L, Townsend E, Buker S, Lancia D (2017). Molecular basis of USP7 inhibition by selective small-molecule inhibitors. Nature.

[26] Davis MI, Pragani R, Fox JT, Shen M, Parmar K, Gaudiano EF, Liu L, Tanega C, McGee L, Hall MD (2016). Small Molecule Inhibition of the Ubiquitin-specific Protease USP2 Accelerates cyclin D1 Degradation and Leads to Cell Cycle Arrest in Colorectal Cancer and Mantle Cell Lymphoma Models. J Biol Chem.

[27] Chauhan D, Tian Z, Nicholson B, Kumar KG, Zhou B, Carrasco R, McDermott JL, Leach CA, Fulcinniti M, Kodrasov MP (2012). A small molecule inhibitor of ubiquitin-specific protease-7 induces apoptosis in multiple myeloma cells and overcomes bortezomib resistance. Cancer Cell.

[28] Duan J, Jin M, Yang D, Shi J, Gao J, Guo D, Tang H, Zhang S, Qiao B (2022). Ubiquitin-specific peptidase 2 inhibits epithelial-mesenchymal transition in clear cell renal cell carcinoma metastasis by downregulating the NF-kappaB pathway. Bioengineered.

[29] Xie H, Zhou J, Liu X, Xu Y, Hepperla A, Simon J, Wang T, Yao H, Liao C, Baldwin A (2022). USP13 promotes deubiquitination of ZHX2 and tumorigenesis in kidney cancer. Proc Natl Acad Sci USA.

[30] Xu Y, Zhu M, Zhang J, Si G, Lv J (2018). Knockdown of ubiquitin-specific peptidase 39 inhibits the malignant progression of human renal cell carcinoma. Mol Med Rep.

[31] Hu W, Su Y, Fei X, Wang X, Zhang G, Su C, Du T, Yang T, Wang G, Tang Z (2020). Ubiquitin specific peptidase 19 is a prognostic biomarker and affect the proliferation and migration of clear cell renal cell carcinoma. Oncol Rep.

[32] Zhou J, Wang T, Qiu T, Chen Z, Ma X, Zhang L, Zou J (2020). Ubiquitin-specific protease-44 inhibits the proliferation and migration of cells via inhibition of JNK pathway in clear cell renal cell carcinoma. BMC Cancer.

[33] Gui D, Peng W, Jiang W, Huang G, Liu G, Ye Z, Wang Y, Xu Z, Fu J, Luo S (2019). Ubiquitin-specific peptidase 46 (USP46) suppresses renal cell carcinoma tumorigenesis through AKT pathway inactivation. Biochem Biophys Res Commun.

[34] Lazaro M, Valderrama BP, Suarez C (2020). de-Velasco G, Beato C, Chirivella I, Gonzalez-Del-Alba A, Lainez N, Mendez-Vidal MJ, Arranz JA: SEOM clinical guideline for treatment of kidney cancer (2019). Clin Transl Oncol.

[35] Patard JJ, Leray E, Rioux-Leclercq N, Cindolo L, Ficarra V, Zisman A, De La Taille A, Tostain J, Artibani W, Abbou CC (2005). Prognostic value of histologic subtypes in renal cell carcinoma: a multicenter experience. J Clin Oncol.

[36] Kodach LL, Peppelenbosch MP (2021). Targeting the Myeloid-Derived Suppressor Cell Compartment for Inducing Responsiveness to Immune Checkpoint Blockade Is Best Limited to Specific Subtypes of Gastric Cancers. Gastroenterology.

[37] Chandran SS, Klebanoff CA (2019). T cell receptor-based cancer immunotherapy: Emerging efficacy and pathways of resistance. Immunol Rev.

[38] Josephs SF, Ichim TE, Prince SM, Kesari S, Marincola FM, Escobedo AR, Jafri A (2018). Unleashing endogenous TNF-alpha as a cancer immunotherapeutic. J Transl Med.

[39] Mollica Poeta V, Massara M, Capucetti A, Bonecchi R (2019). Chemokines and Chemokine Receptors: New Targets for Cancer Immunotherapy. Front Immunol.

[40] Lipson EJ (2013). Re-orienting the immune system: Durable tumor regression and successful re-induction therapy using anti-PD1 antibodies. Oncoimmunology.

[41] Larkin J, Chiarion-Sileni V, Gonzalez R, Grob JJ, Cowey CL, Lao CD, Schadendorf D, Dummer R, Smylie M, Rutkowski P (2015). Combined Nivolumab and Ipilimumab or Monotherapy in Untreated Melanoma. N Engl J Med.

[42] Braun D, Bakouny Z, Hirsch L, Flippot R, Van Allen E, Wu C, Choueiri T (2021). Beyond conventional immune-checkpoint inhibition - novel immunotherapies for renal cell carcinoma. Nat Rev Clin Oncol.

[43] Sternberg CN, Davis ID, Mardiak J, Szczylik C, Lee E, Wagstaff J, Barrios CH, Salman P, Gladkov OA, Kavina A (2010). Pazopanib in locally advanced or metastatic renal cell carcinoma: results of a randomized phase III trial. J Clin Oncol.

[44] Escudier B, Eisen T, Stadler WM, Szczylik C, Oudard S, Siebels M, Negrier S, Chevreau C, Solska E, Desai AA (2007). Sorafenib in advanced clear-cell renal-cell carcinoma. N Engl J Med.

[45] Motzer RJ, Hutson TE, Tomczak P, Michaelson MD, Bukowski RM, Rixe O, Oudard S, Negrier S, Szczylik C, Kim ST (2007). Sunitinib versus interferon alfa in metastatic renal-cell carcinoma. N Engl J Med.

[46] Hutson TE, Escudier B, Esteban E, Bjarnason GA, Lim HY, Pittman KB, Senico P, Niethammer A, Lu DR, Hariharan S (2014). Randomized phase III trial of temsirolimus versus sorafenib as second-line therapy after sunitinib in patients with metastatic renal cell carcinoma. J Clin Oncol.

[47] Gao M, Guo G, Huang J, Kloeber J, Zhao F, Deng M, Tu X, Kim W, Zhou Q, Zhang C (2020). USP52 regulates DNA end resection and chemosensitivity through removing inhibitory ubiquitination from CtIP. Nat Commun.

