# Supplementary material for: A risk signature of ubiquitin-specific protease family predict the prognosis and therapy of kidney cancer patients
Source: BMC Nephrol. 2023 May 31;24:153. doi: 10.1186/s12882-023-03215-0 (PMC10230779; doi:10.1186/s12882-023-03215-0)
Supplement: Supplementary file 2 — Additional file 2: Figure S1. Validation of PRUSPs risk signature in external datasets. (A) The Kaplan-Meier survival curves of high-/low-PRUSPs-risk groups in the E-MTAB-1980 dataset. AUC value of time-dependent ROC curves demonstrates the prognostic capacity of PRUSPs risk signature. The dot-plot was depicted to represent the OS, OS status, and risk score in the E-MTAB-1980 dataset. The heatmap showed the expression of PRUSPs in high and low-risk groups of the E-MTAB-1980 dataset. (B) The Kaplan-Meier survival curves of high-/low-PRUSPs-risk groups in the TCGA-KIRP dataset. AUC value of time-dependent ROC curves demonstrates the prognostic capacity of PRUSPs risk signature. The dot-plot was depicted to represent the OS, OS status, and risk score in TCGA-KIRP dataset. The heatmap showed the expression of PRUSPs in high and low-risk groups of the TCGA-KIRP dataset. Figure S2. DCA analysis of different combination based clinical variables. [file 12882_2023_3215_MOESM2_ESM.pptx]

## Slide 1
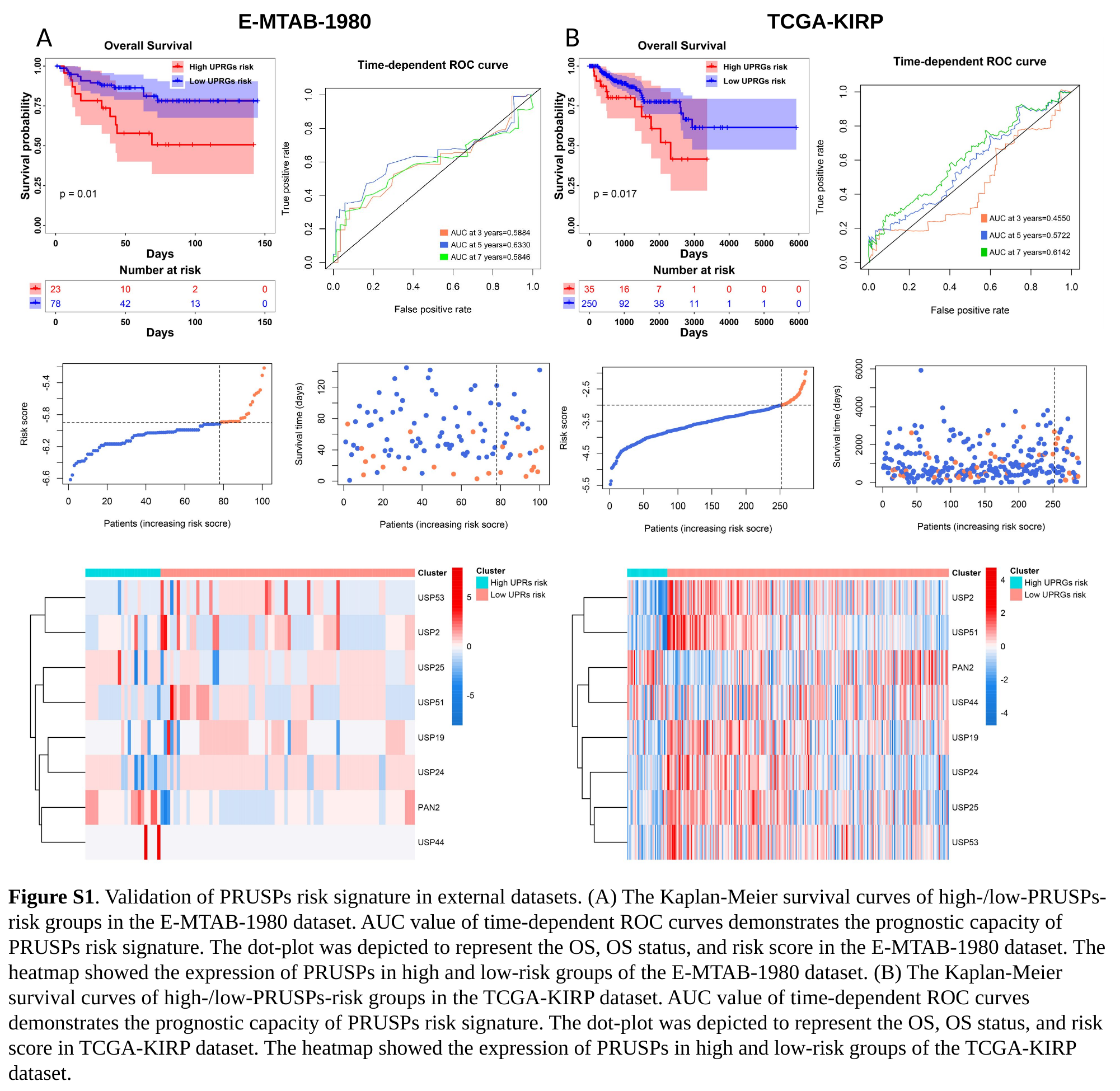

E-MTAB-1980
TCGA-KIRP
A
B
Figure S1. Validation of PRUSPs risk signature in external datasets. (A) The Kaplan-Meier survival curves of high-/low-PRUSPs-risk groups in the E-MTAB-1980 dataset. AUC value of time-dependent ROC curves demonstrates the prognostic capacity of PRUSPs risk signature. The dot-plot was depicted to represent the OS, OS status, and risk score in the E-MTAB-1980 dataset. The heatmap showed the expression of PRUSPs in high and low-risk groups of the E-MTAB-1980 dataset. (B) The Kaplan-Meier survival curves of high-/low-PRUSPs-risk groups in the TCGA-KIRP dataset. AUC value of time-dependent ROC curves demonstrates the prognostic capacity of PRUSPs risk signature. The dot-plot was depicted to represent the OS, OS status, and risk score in TCGA-KIRP dataset. The heatmap showed the expression of PRUSPs in high and low-risk groups of the TCGA-KIRP dataset.

## Slide 2
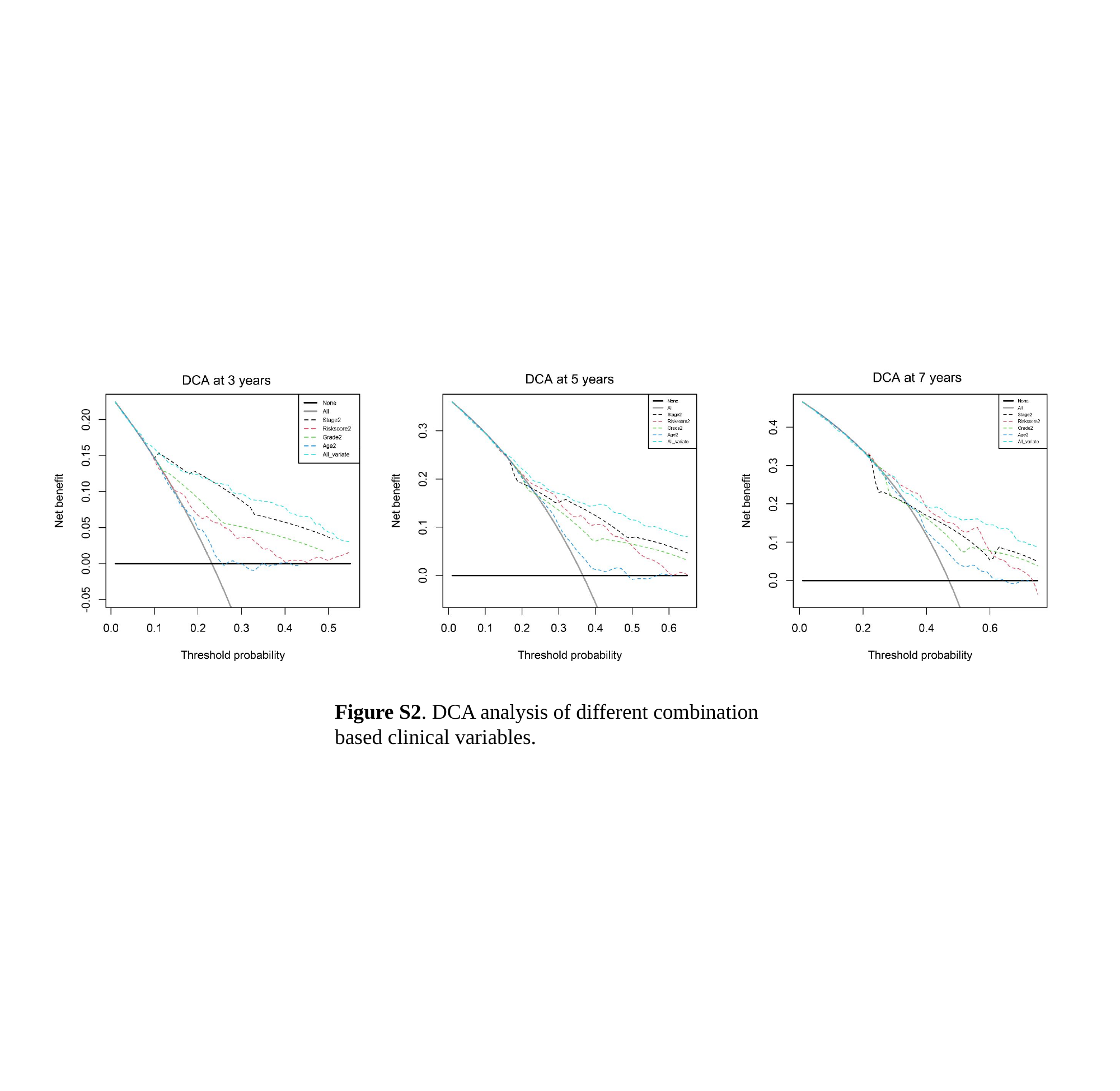

Figure S2. DCA analysis of different combination based clinical variables.
